# Development of Implantable Wireless Sensor Nodes for Animal Husbandry and MedTech Innovation

**DOI:** 10.3390/s18040979

**Published:** 2018-03-26

**Authors:** Jian Lu, Lan Zhang, Dapeng Zhang, Sohei Matsumoto, Hiroshi Hiroshima, Ryutaro Maeda, Mizuho Sato, Atsushi Toyoda, Takafumi Gotoh, Nobuhiro Ohkohchi

**Affiliations:** 1 Research Center for Ubiquitous MEMS and Micro Engineering (UMEMSME), National Institute of Advanced Industrial Science and Technology (AIST), Namiki 1-2-1, Tsukuba 305-8564, Japan; chou-ran@aist.go.jp (L.Z.); daihou.chou@aist.go.jp (D.Z.); sohei.matsumoto@aist.go.jp (S.M.); hiroshima-hiroshi@aist.go.jp (H.H.); maeda-ryutaro@aist.go.jp (R.M.); 2 College of Agriculture Ibaraki University, Chuo Ami Inashiki 3-21-1, Mito 300-0393, Japan; mizuho.sato.0519@gmail.com (M.S.); atsushi.toyoda.0516@vc.ibaraki.ac.jp (A.T.); 3 Kuju Agricultural Research Center, Graduate School of Agriculture, Kyushu University, Fukuoka 878-0201, Japan; gotoh@agri.kagoshima-u.ac.jp; 4 Department of Agricultural Sciences and Natural Resources, Kagoshima University, Korimoto 1-21-24, Kagoshima 890-0065, Japan; 5 Faculty of Medicine, University of Tsukuba, Tennoudai 1-1-1, Tsukuba 305-8577, Japan; nokochi3@md.tsukuba.ac.jp

**Keywords:** implantable device, wireless sensor node, RFID, remote charge, animal husbandry, artificial magnetic field, MedTech innovation

## Abstract

In this paper, we report the development, evaluation, and application of ultra-small low-power wireless sensor nodes for advancing animal husbandry, as well as for innovation of medical technologies. A radio frequency identification (RFID) chip with hybrid interface and neglectable power consumption was introduced to enable switching of ON/OFF and measurement mode after implantation. A wireless power transmission system with a maximum efficiency of 70% and an access distance of up to 5 cm was developed to allow the sensor node to survive for a duration of several weeks from a few minutes’ remote charge. The results of field tests using laboratory mice and a cow indicated the high accuracy of the collected biological data and bio-compatibility of the package. As a result of extensive application of the above technologies, a fully solid wireless pH sensor and a surgical navigation system using artificial magnetic field and a 3D MEMS magnetic sensor are introduced in this paper, and the preliminary experimental results are presented and discussed.

## 1. Introduction

Biotelemetry has been developed over a number of decades for the remote gathering of various vital signs from animals and ambulatory patients by introducing telemetry in biological, medicinal, and other medical instruments. Historically, one of the most impressive and significant advances came during the Space Race between 1950 and 1960 for the investigation of physiological effects during exposure to zero-gravity [1]. Recently, the research interests and application focus of Wireless Sensor Networks (WSN) and the Internet of Things (IoT) have been moving from infrastructure monitoring [2,3] to biotelemetry for healthcare and medical care [4,5]. The explosive growth of and dramatic progress in diagnostic techniques, smart sensing technologies, and artificial intelligence (AI) have inaugurated a new era in biotelemetry, which isn’t focused solely on those who are sick, but is about keeping people healthy and improving health [6,7]. Particularly in most of the developed countries, remote diagnosis and remote medical treatment by telemetry have become very attractive and essential options for dealing with insufficient human labor and other social problems resulting from the aging tendency of the population.

Among the different types of devices used for biotelemetry, implantable devices can accumulate more accurate data while experiencing fewer effects resulting from the surrounding environment and wearing method, it is therefore believed to be more effective than using wearable devices for disease prevention and disease diagnosis in early stages. Related works have been published on biotelemetry for the monitoring of intravascular pressure [8], blood pressure [9], and conditional bladder neuromodulation [10]. Additionally, implantable devices can be used to monitor biological information of animals for the development of new food and new medicine for the improvement of animal husbandry, as well as for MedTech innovation [11]. Compared to traditional wireless sensors, which include appropriate microelectromechanical systems (MEMS) for the signals to be monitored, the use of RF transceivers for data communication and batteries to power the system face significant challenges associated with implantable devices. These challenges include system scale-down (both physically and electrically), flexible assembly of modules and components, power supply, and packaging of the system in bio-compatible material with a sufficient lifetime. In addition to ethical and privacy issues, the practical application of implantable devices also requires great and continuous effort in the areas of advanced system design and packaging technologies to reduce their perioperative risks to the lowest possibility and to avoid any associated comorbidities.

In our previous works, we have developed one of the world’s smallest and lowest-power wireless sensor nodes for environmental monitoring [12,13]. An all-in-one packaged sensor node using low-temperature co-fired ceramic (LTCC) is also under development for the maintenance-free monitoring of highways, bridges, tunnels, and many other infrastructural elements [14]. In further work on implantable devices, a radio frequency identification (RFID) chip with a hybrid interface that is able to communicate with both RFID Reader/Writer and MCU was introduced into the field of sensor nodes for the first time. The RFID makes it possible to use RF-Transmitters instead of RF-Transceivers in order to save the majority of the power consumed by communication while the measurement mode, as well as power ON/OFF, can be controlled by RFID reader/writer even after implantation. In addition, a remote wireless and high efficient power transmission system with an improved accessibility distance of up to 5 cm was successfully developed. This enables the power accelerometer, pH sensor [15], and electromyogram (EMG) to have a significantly extended lifetime for use in many other applications.

In this paper, we summarize our previous related works, as well as our latest results on the development, evaluation, and application of wireless sensor nodes and power transmission systems for animal monitoring, as well as for MedTech innovation. Field tests were carried out using laboratory mice to challenge the minimum size of the sensor node, and a cow to explore the performance of the sensor node, as well as its remote power transmission system. In addition, a fully solid wireless pH sensor and a surgical navigation system with a spatial resolution of several millimeters was developed using an artificial magnetic field and 3D MEMS magnetic sensor node for further application of the above technologies in MedTech innovation. The motivation of this paper is to offer practically applicable information and technical details to as many as possible and to the related community for the promotion of such applications. Additionally, we believe that this paper may also help those who are in the medical field to understand what the sensor community is able to offer through close collaborations.

## 2. Design, Assembly and Package

### 2.1. Wireless Sensor Nodes

A wireless sensor node is generally composed of MCU, RFIC, antenna and its matching circuit, battery, and various sensors, depending on the application. Since most implantable devices require small and flexible wire to perform sensing and, in some cases, stimulation [16], it is difficult to integrate sensors with ICs by monolithic or heterogeneous processes. Therefore, in this work, a fully-integrated RFIC transmitter with embedded 8051-MCU and general I/O interfaces was selected to reduce the component count to the lowest number possible. Even though a high carrier frequency such as 2.4 GHz enables us to use small-chip antennas, microstrip antennas, or other novel antennas [17], a low carrier frequency such as 400–950 MHz is preferable for implantable devices due to their low return-loss from the skin [18]. In this work, the carrier frequency of the sensor node was therefore set at 923 MHz. An 8.3-cm-long dipolar antenna was used for data communications.

**Figure 1:**
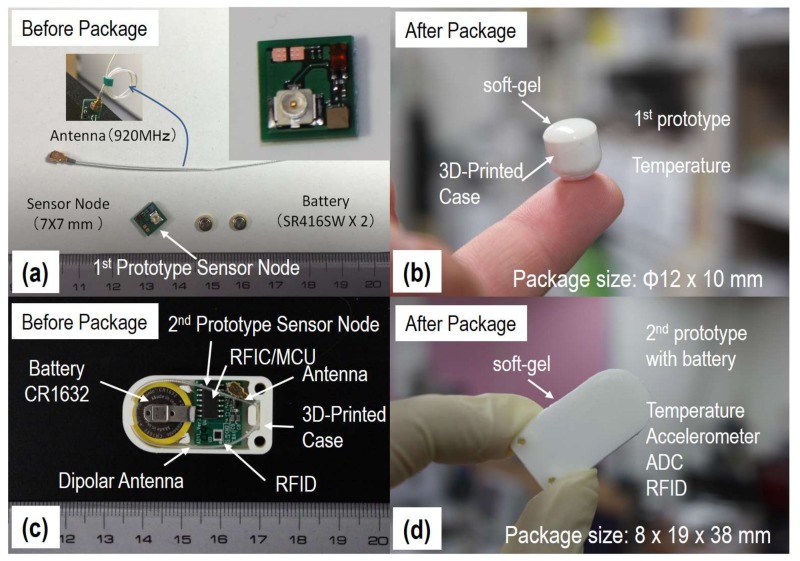
Photos of the first prototype sensor node and second prototype sensor node before (**a**,**c**) and after packaging (**b**,**d**) using the 3D-printed case, and then covering and filling with bio-compatible soft-gel.

A digital temperature sensor was used to monitor body temperature. As shown in Figure 1a, the size of our first prototype sensor node was as small as 7 (W) × 7 (L) × 3.1 (H) mm, including the temperature sensor, fully-integrated RFIC, passive components, and antenna-matching circuit. All the components were first installed with the antenna and power supply in a polylactic acid (PLA) case made by 3D printer, and then a bio-compatible (silicon, etc.) soft-gel was used to fill in the PLA case to protect those components from bio-contamination, as well as short-circuit by blood. After packaging, the size had increased to φ12 (D) × 10 (H) mm, as shown in Figure 1b, and the weight was 1.6 g. Normally, the size and weight of laboratory mice are 10–15 cm and 15–20 g, respectively. Therefore, the packaged sensor node was believed to be potentially applicable to laboratory mice. In addition, experimental results revealed that no obvious degradation to antenna efficiency could be found after packaging, which indicates good material and structural feasibilities.

The lifespan of the power supply is one of the most critical issues for implantable sensors. In this work, an RF transmitter was used instead of a transceiver to dramatically reduce standby current from a few mA to a few μA. Since on-duty current of the developed wireless sensor node was 20–30 mA and the duty-cycle was expected to be between 1% to 0.01% (measurement duration was within a few tens of ms, interval time was set at a few minutes), the RF transmitter enabled us to save power consumption by several orders. As a result, a hybrid RFID chip (MAGICSTRAP^®^, Murata Manufacturing Co., Ltd., Nagaokakyo, Japan) was introduced in our second prototype sensor node, as shown in Figure 1c. The hybrid RFID has I2C digital interface, which means its memory can be accessed by both RFID reader/writer and MCU to control ON/OFF, interval time, and measurement mode. In the second prototype sensor node, we also included an accelerometer to monitor the activities and posture of animals, an ADC to measure the voltage of power supply, a general I/O, and a digital interface for pH sensing and many other applications. The size of the sensor node was 12 (W) × 16 (L) × 5 (H) mm. After packaging with the antenna and the CR1632 battery, the size had increased to 19 (W) × 38 (L) × 8 (H) mm, as shown in Figure 1d, and the weight was 8.6 g.

The lifetime of the battery can be calculated from the theoretical power consumption of each sensor node. However, it may be slightly affected by the peak current of the sensor nodes, working temperature, and other factors. Therefore, Table 1 lists the measured lifetime of the battery and the calculated power consumption under various conditions. To further decrease the power, a low frequency (10–30 Hz) is used, and a self-powered MEMS vibration sensor is under development to replace the accelerometer [19]. The results are encouraging and will be published elsewhere.

### 2.2. Wireless Power Transmission System

**Figure 2:**
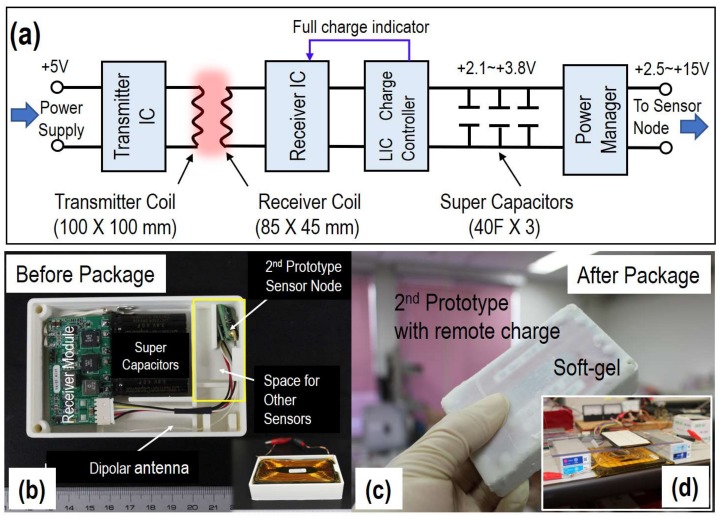
Schematic view of the wireless power transmission system, which includes a power transmitter module and power receiver module (**a**); photos of the second prototype sensor node with wireless power receiver module before (**b**) and after packaging (**c**); and photo of the evaluation system (**d**).

Impressive work on kinetic and infrared energy harvesting devices has been reported and is expected for implantable applications [20,21]. However, the achievable power density, a few μW/cm^2^, is far from the mW/cm^2^ required by most applications. Figure 2a shows the system block of our wireless power transmission system. It consists of a wireless power transmitter module and a wireless power receiver module. The wireless power receiver module has lithium-ion capacitors (LIC) for quick power storage, a high-efficiency LIC charge unit, and a DC-DC converter for power management. The details have been discussed and published elsewhere [14].

In this work, three pcs of LIC with a total capacitance of 120 F were used for energy storage due to their low self-discharge, their wide operating temperature of −25~+85 °C, and especially their rapid charge capability. A power backup circuit made it possible to supply 3 V to the sensor node when the voltage of the LIC was between 2.1 V and 3.8 V. To avoid any interference, power transmission is disabled when the sensor module is ready to send measured data. To protect the LIC from over charge and over discharge, wireless power receiver module is disabled if the voltage of the LIC reaches 3.8 V, and the output is shut off if the voltage of the LIC deceases to 2.1 V. Thanks to the optimized matching circuits and the customized transmitter and receiver coils, power transmission distance can be as large as 5 cm, while keeping a certain level of efficiency, such as >50%, as shown in Figure 2d. This enables full-charge of those LICs in a few minutes if the supplied current to the transmitter is 1 A at a voltage of 5 V.

Figure 2b,c shows photos of the assembled second prototype sensor node with wireless power module before and after packaging, respectively. The size of the module was 60 (W) × 100 (L) × 20 (H) mm, and the weight was 190 g. Although both the size and the weight are not suitable for small husbandry animals, its performances can be evaluated by using wagyu (Japanese cow) after implanting them underneath the skin, which may give us valuable information for further development. In addition, it is believed that the size and the weight could be dramatically reduced by advances in LIC technology, as well as optimization of the coil. Our latest results also show that the receiver coil can be reduced to about half size without a significant decrease in power transmission distance. Those results will be presented in our future publications.

## 3. Results and Discussion

### 3.1. Temperature Varitions of Laboratory Mice

Rodents play an invaluable role in biomedical research, since approximately 95% of all laboratory animals are mice and rats. Mice have less than half of the body weight of rats and require a much lower feeding and maintenance cost than rats. However, due to the difficulties of monitoring biological information of laboratory mice in real time by using biotelemetry, roughly 50% more scientific research studies have been done on laboratory rats than on laboratory mice. By using the first prototype sensor node, as shown in Figure 1b, implant experiments were successfully carried out on laboratory mice to monitor their body temperatures.

**Figure 3:**
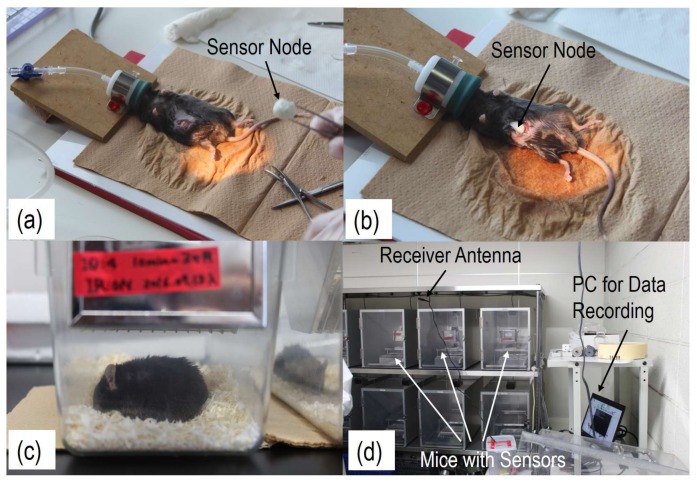
Photos of a mouse before (**a**), during (**b**), and after (**c**) implantation. Then, several mice were kept in cages to capture their body temperatures every few minutes (**d**).

Figure 3 shows photos of the implant experiments and the monitoring system. The sensor node was implanted into the abdominal cavity of the mouse, due to the size of the sensor nodes. No deterioration of data transmission was found after the implantation due to the low return-loss from the skin or the interactions between body and antenna [22]. It is worth noting that the lifetimes of the CR1025 battery were measured at 28 days and 27 days, when the interval times were set at 3 min and 10 min, respectively. Clearly, this is the same regardless of actual power consumption, and is much shorter than the tested lifetime in laboratory, which is shown in Table 1. This is believed to be due to the elevated battery temperature of up to 38 °C after implantation.

**Figure 4:**
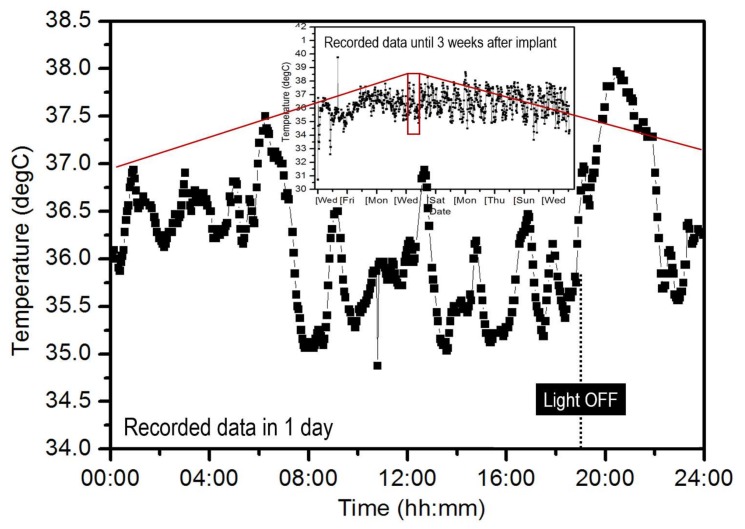
Recorded body temperature of a mouse in a day. The inset is the data for the three weeks directly following the implantation.

Figure 4 shows the recorded body temperature of a mouse at one day and at three weeks after implantation. It may take about a week to recover from the surgery. The body temperature of the mouse fluctuated surprisingly between 34.5 °C and 38.5 °C in a single day and stayed high at night when the mouse was active after turning off the room lights. E. M. Bradford et al. concluded that the body temperature of a mouse is closely related to its heart-rate [23]. Therefore, the inter-activities of several mice in the same cage can be recorded by the developed sensor nodes. This is close to their natural ecology and is believed to be of significant important. The above results clearly indicate that the developed sensor node enables us to use mice instead of rats for new food and medicine development by dramatically reducing the running cost and improving the data accuracy.

**Figure 5:**
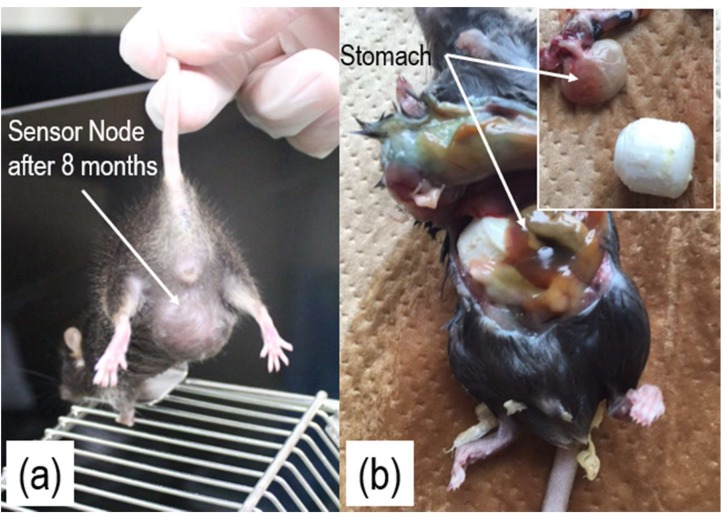
Photo of a mouse eight months after the implantation (**a**), and photos of dissection after the mouse had been found dead a few days after the implantation (**b**).

As shown in Figure 5, some mice survived for more than eight months after the implantation, while a few were dead a couple of days after the implantation. In general, gel has poor water permeability resistance. Figure 5a clearly indicates that water permeability resistance was improved after using both the PLA case and gel, since no clear deuteriation was found even after eight months. However, further evaluation is necessary if we are to expect a few years of implantation. As shown in Figure 5b, the results of dissection suggest that the cause of death was intestinal obstruction. In addition to selecting a suitable location for implantation, further scale-down of both size and weight of the sensor nodes, especially the antenna module, may help reduce the life-risk of the mouse during experiments. Among state-of-the-art technologies, stacked antennas or printed antennas would be potential candidates if efficiency and bandwidth could be improved [24].

### 3.2. Monitoring of Wagyu for Animal Husbandry

**Figure 6:**
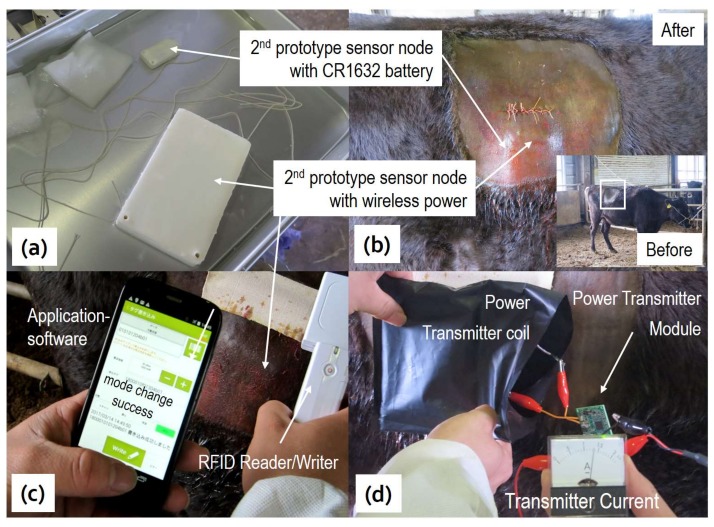
Photos of the sensor nodes ready for implant (**a**); the cow after implant (**b**); remote control test (**c**); and remote wireless charge test (**d**).

Japanese cow, which is also called wagyu, is raised in a luxurious fashion with specifically tailored diets and lifestyles. Therefore, real-time and long-term monitoring of their health conditions, as well as their daily activities, is essential to ensuring quality of beef, as well as to reducing the risk of sudden death [25]. As shown in Figure 6a,b, sensor nodes with battery power supplies (as shown in Figure 1d) and wireless power modules (as shown in Figure 2c) were implanted under the skin of a wagyu at a location close to the rumen to evaluate their performances, as well as to investigate the effects of rumen movement.

In contrast to the laboratory mice, it was found that data transmission distance was reduced from 30 m to 10 m after the implantation. This is believed to be due to absorption by surrounding blood, direction of the antenna, and a layer of skin and fat more than 2 cm thick. Before implantation, the RFID chip could be accessed by RFID writer if the distance between them was less than 6 cm, and the writing power was set at 1 watt. The photo in Figure 6c demonstrates that if the distance between RFID writer and cow was less than 4 cm, the measurement mode and power ON/OFF could be successfully switched. It is noteworthy that the frequency of the RFID writer was 920 MHz, which is the same as the frequency range of RFIC used for data communication. The results clearly suggested that a directional antenna as used by RFID is less affected by the surroundings. Moreover, the photo in Figure 6d shows that wireless power transmission was successfully carried out at 1.5 A charge current and 5 V supplied voltage. The distance between the power transmitter coil and the power receiver coil was estimated at 4 cm to 5 cm at a carrier frequency of 110 kHz, which is the same as the results measured in the laboratory [14].

**Figure 7:**
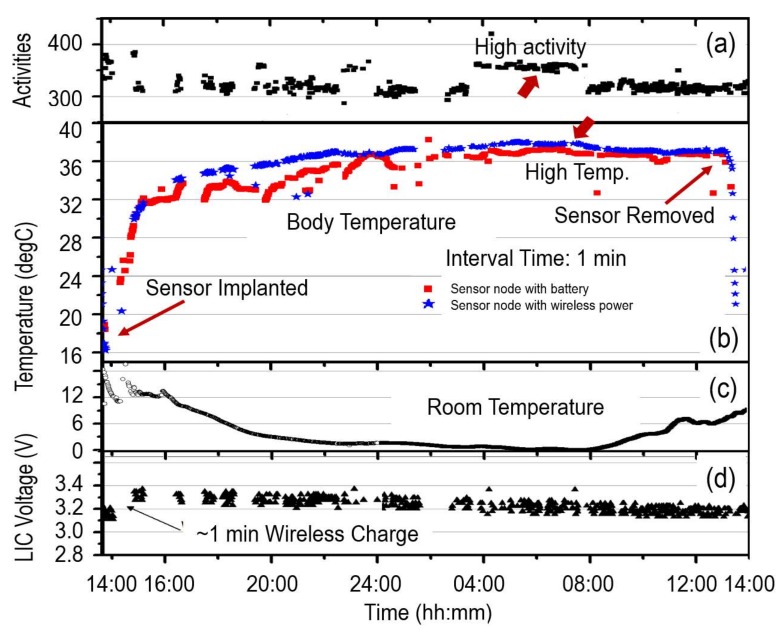
Evaluation results after implanting the second and third prototype sensor nodes into a wagyu (Japanese cow): calculated activities form the measured data of the accelerometer (**a**); measured body temperature (**b**); measured room temperature (**c**); and measured voltage of the LIC (**d**) for the duration of a day after implantation.

The recorded data is summarized in Figure 7 for comparison. It can be seen that the sensor had stabilized only a few hours after implantation. The body temperature shown in Figure 7b,c reveals that the sensor node that uses a battery as power supply and faces toward the outside of the body, is more influenced by room temperature than the sensor facing toward the inside of the body. However, temperature variation is quite limited compared to that of the laboratory mice. Even though the measured body temperature showed sufficiently high accuracy, the relationship between activities and the body temperature of the cow was hardly recognized. These results and phenomena reveal the effects of the differing physical constitutions to different animals.

The activity of the cow measured by using an accelerometer in Figure 7a revealed that preferred biological information, such as activities and postures, can be effectively monitored by the developed sensor nodes at the cost of increased power consumption by orders. Figure 7d reveals that the voltage of LIC was increased from 3.15 V to 3.35 V after about a one-minute remote charge and then decreased to 3.2 V after data acquisition and collection for 24 h. The results indicate that the sensor node may survive for one day at the cost of a 0.15 V LIC voltage decrease. Therefore, it could survive for two weeks if the LIC was fully charged from 2.1 V to 3.8 V. The use of LIC instead of a rechargeable battery dramatically reduces the charge time from hours to minutes, thus dramatically improving the efficiency of human labor.

**Figure 8:**
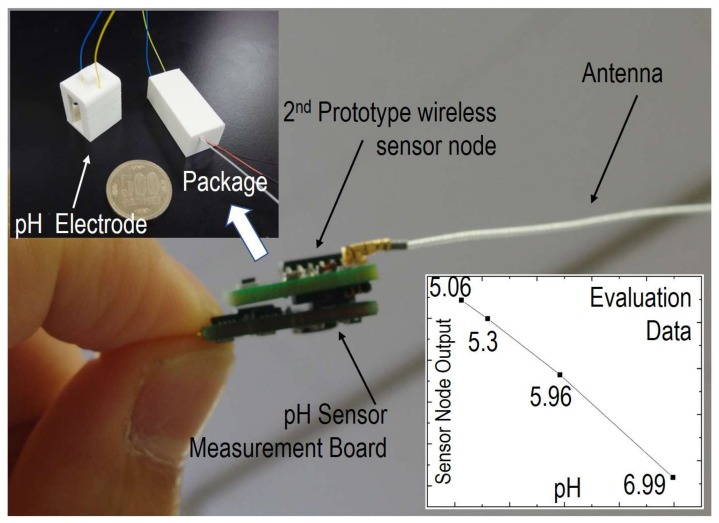
Photos of the developed fully solid wireless pH sensor before and after packaging. The inset shows the evaluation results collected by the wireless receiver.

To improve the quality of beef, another essential biological data point is the rumen pH value. In our previous work, a fully solid-type pH sensor was developed to monitor gastric acid in rumen in real time [15]. Figure 8 shows a photo of the miniaturized wireless pH sensor assembled with the second prototype sensor node. The on-board temperature sensor can be used to calibrate the sensor for dealing with temperature drift, and the on-board accelerometer is useful for simultaneously recording rumen motility. The results in the inset of Figure 8 clearly demonstrate the high accuracy and good linearity of the measured pH value. Further development, especially on system integration and packaging with the second prototype sensor node, is ongoing for the long-term monitoring of reflux in the esophagus of aged people.

### 3.3. Positioning for Surgical Navigation

Due to the advances in electronic devices and diagnostic techniques, laparoscopic surgery has been in use for decades to reduce body cavity invasion. However, even with the benefits of 3D vision and simulation, the risk of fatal bleeding is still high and inevitable due to the misunderstanding of anatomical location and the misslocation of surgical tools [26,27].

**Figure 9:**
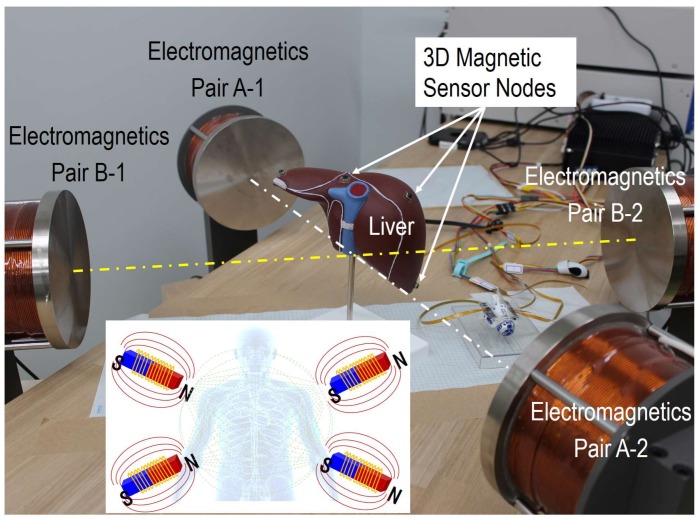
Photos and mechanism of the positioning system by using 3D magnetic sensors and artificial magnetic fields.

As a further application of implantable devices, a 3D magnetic sensor was included in the sensor node and then attached to organs or surgical instruments, where an artificial magnetic field was created with a unique strength or direction for each location. Figure 9 shows a photo of our developed system for the feasibility study, and the inset shows its mechanism. The strength of the magnetic field was designed to be between 1 mT and 30 mT, with a total volume of 60 × 60 × 60 cm^3^, and the sensitivity of the 3D magnetic sensor was 1.1 μT. Several pairs of electromagnetics with fixed driving frequencies from DC to a few Hz were designed to recognize the individual magnetic fields generated by each pair and location, while the rotation of the sensor nodes can also be calculated, even at a low sampling rate of a few tens of Hz, to reduce its power consumption. Preliminary results suggest that a positioning accuracy of 3–5 mm can be achieved based on the raw data from the sensors, and can then be improved to 2–3 mm after using the digital filter. The location data of each sensor will be imported into 3D simulated human liver models to upgrade the system from simulation to navigation for MedTech innovations.

While this work is not directly related to the above sensor node prototypes developed in Figure 1 and Figure 2, it may deliver information to people outside the sensor community that MedTech innovation could be greatly enhanced by sensing technologies. This work is still ongoing, and the details will be published in the near future.

## 4. Conclusions

This paper summarized our previous and latest works on the development, evaluation, and application of wireless sensor nodes and power transmission systems for animal monitoring, as well as for MedTech innovation from system scale-down and application points of view. The results indicated that measurement mode and power ON/OFF can be remote controlled even after implant, which enables us to use transmitters instead of transceivers to reduce the power consumption by orders. The combination of wireless power transmission and LIC greatly extend the lifespan of the sensor node, which has a user-friendly interface. Moreover, our preliminary results strongly suggested that MedTech may certainly benefit from the wireless sensor node and biotelemetry innovations. The results are encouraging and promising at the current stage, while further development of various self-powered and low-power sensors for biological monitoring is extremely important, in addition to big data collection and analysis. We believe this paper may also help those who are in the medical field to understand what the sensor society can do if we have close collaborations.

## Figures and Tables

**Table 1:** Measured battery lifetime and calculated average power consumption of the developed sensor nodes under various conditions.

Sensor Node	Interval Time	Battery	Meas. Lifetime	Cal. Power Consumption
1st Prototype Temperature	10 min	SW612	3 weeks	24 μW
	10 min	CR1025	16 weeks	
2nd Prototype Temperature Accelerometer/Voltage Hybrid RFID	1 min	CR1632	12 weeks	600 μW
	10 min	CR1632	31 weeks	230 μW
	1 min	Remote-charge		600 μW
